# Open Burn Pit Exposure in Headache Disorder and Migraine

**DOI:** 10.1001/jamanetworkopen.2024.31522

**Published:** 2024-09-04

**Authors:** Jason J. Sico, Sarah E. Anthony, Manali Phadke, Kaicheng Wang, Melissa Skanderson, John P. Ney, Elizabeth K. Seng, Robert E. Shapiro, Friedhelm Sandbrink, Joel D. Scholten, Glenn D. Graham, Sharyl R. Martini, Brenda T. Fenton

**Affiliations:** 1 Research, Education, Evaluation and Engagement Activities Center for Headache, Headache Centers of Excellence, US Department of Veterans Affairs (VA), Orange, Connecticut; 2 Department of Neurology, Yale School of Medicine, New Haven, Connecticut; 3 Headache Center of Excellence, VA Connecticut Healthcare System, West Haven; 4 Department of Internal Medicine, Yale School of Medicine, New Haven, Connecticut; 5 Yale Center for Analytic Sciences, Yale School of Public Health, New Haven, Connecticut; 6 Department of Neurology, Boston University School of Medicine, Boston, Massachusetts; 7 Ferkauf Graduate School of Psychology, Yeshiva University, Bronx, New York; 8 Montefiore Headache Center, Montefiore Medical Center, Bronx, New York; 9 Department of Neurology, Albert Einstein College of Medicine, Bronx, New York; 10 Department of Neurology, University of Vermont Medical Center, Burlington; 11 Division of Pain Management, Department of Neurology, VA Medical Center, Washington DC; 12 Department of Physical Medicine and Rehabilitation, VA, Washington, DC; 13 Neurology Program Office, Veterans Health Administration, Washington, DC; 14 Department of Neurology, University of California, San Francisco; 15 Department of Neurology, Baylor Medical School, Houston, Texas; 16 Pain Research, Informatics, Multi-morbidities, and Education Center, VA Connecticut Healthcare System, West Haven

## Abstract

**Importance:**

Open burn pits have commonly been used for waste disposal by the US military but have not been systematically investigated as an independent risk factor for headache disorders.

**Objective:**

To evaluate the association between exposure to open burn pits and incidence of headache and migraine.

**Design, Setting, and Participants:**

This retrospective cohort study used data from the Veterans Health Administration Headache Cohort along with data from the US Department of Defense and the Airborne Hazards and Open Burn Pit (AH&OBP) Registry to assess registry participants with potential exposure to open burn pits in the Veterans Health Administration from April 1, 2014, through October 31, 2022. Participants were included by linking data from the AH&OBP Registry to their US Department of Defense and Veterans Health Administration electronic health records. Those with preexisting headache were removed from the analytic sample. The analysis was conducted between November 1, 2022, and January 31, 2024.

**Exposure:**

Open burn pit exposure composite variables based on the registry questionnaire were examined, specifically being near open burn pits, days near open burn pits, and having open burn pit duties.

**Main Outcomes and Measures:**

Primary incident outcomes included medically diagnosed headache disorders and medically diagnosed migraine.

**Results:**

The analytic sample included 247 583 veterans (mean [SD] age, 27.9 [7.7] years; 222 498 [89.9%] male). After covariates were controlled for at baseline, participants who were near an open burn pit with open burn pit duties had the highest adjusted odds of medically diagnosed headache disorders (adjusted odds ratio [AOR], 1.59; 95% CI, 1.46-1.74), migraine (AOR, 1.60; 95% CI, 1.43-1.79), and self-reported disabling migraine (AOR, 1.93; 95% CI, 1.69-2.20) compared with those without exposure. The 2 highest quartiles of cumulative burn pit exposure (290-448 days and >448 days) had significantly higher adjusted odds of medically diagnosed headache (290-448 days: AOR, 1.20; 95% CI, 1.09-1.31; >448 days: AOR, 1.55; 95% CI, 1.41-1.70) and migraine (290-448 days: AOR, 1.19; 95% CI, 1.07-1.34; >448 days: AOR, 1.48; 95% CI, 1.32-1.65).

**Conclusions and Relevance:**

In this cohort study, a dose-dependent association existed between open burn pit exposure and medically diagnosed headache and migraine. These new data identify potentially important associations between open burn bit exposure and new-onset headache among service personnel as well as a possible health condition that may be encountered more frequently in Veterans Health Administration facilities during mandatory screening for military exposures.

## Key Points

**Question:**

Is there an association between open burn pit exposure and incident headache and migraine?

**Findings:**

In this cohort study, 247 583 veterans with both forms of exposure to open burn pits (who were near sites and had open burn pit duties) had the highest risk of medically diagnosed headache disorders, migraine, and self-reported disabling migraine compared with those without exposure.

**Meaning:**

Increasing levels of open burn pit exposure may lead to an increased risk of incident headache and migraine among former service members.

## Teaser

This cohort study of veterans examines whether open burn pit exposure is associated with incident headache and migraine.

## Introduction

An increasing body of evidence suggests that airborne hazards may be a risk factor for headache disorders, including among 9/11 World Trade Center (WTC) collapse survivors and first responders.^1,2,3,4,5^ Airborne hazard refers to any chemical, physical, or biological agent that is inhaled. Open burn pits have been used for waste disposal by the US military in Iraq, Afghanistan, and other areas of the Southwest Asia theater of military operations.^6,7,8^ Smoke and fumes from open burn pits are thought to contain toxic compounds, such as polycyclic aromatic hydrocarbons, volatile organic compounds, and heavy metals.^9,10,11^

In 2013, the US Congress enacted Public Law 112-260 (eAppendix 1 in Supplement 1), which directed the US Department of Veterans Affairs (VA) to establish the Airborne Hazards and Open Burn Pit (AH&OBP) Registry for service members who may have been exposed to airborne hazards produced by open burn pits in hopes of understanding whether open burn pit exposure may be a source of lasting injury to current and former service members.^9,12^ The Sergeant First Class Heath Robinson Honoring our Promise to Address Comprehensive Toxicities Act of 2022 (ie, Honoring our PACT Act) expanded health care and benefits for veterans exposed to open burn pits, Agent Orange, and other toxins.^13^

Although airborne hazard exposure has been associated with headache disorders in other populations,^1,2^ whether airborne hazard exposure is associated with new-onset headaches among active service members and veterans has not been explored. To address this gap in our understanding and recognizing the potential health and health care implications for service members and veterans, the US Congress directed the Veterans Health Administration (VHA) to investigate whether relationships between open burn pit exposure and headache disorders exist.^14^ Among a cohort of current and former service members who served in Operations Desert Storm/Desert Shield, New Dawn, or Iraqi Freedom/Enduring Freedom and were free of headache disorders before deployment,^15^ we report on associations between the presence and duration of open burn pit exposure and incident headache and migraine.

## Methods

### Study Oversight, Reporting, and Protocol Approval

This cohort study used AH&OBP Registry data in combination with electronic health record (EHR) data from the US Department of Defense (DOD) and the VHA to examine the burden of headache among the registry participants and its association with exposure to open burn pits. The VHA Headache Centers of Excellence research team sent a formal request for registry data to the AH&OBP Center of Excellence. Variables used from the AH&OBP Registry include those related to open burn pit exposure and duties, branch of military service, and functional impairment related to self-reported migraine (eAppendix 2 in Supplement 1). The study was approved by the VA Connecticut Healthcare System Institutional Review Board, which approved a waiver of informed consent because this was deemed as a minimal risk study and the research could not practically be performed without the waiver. Reporting followed the Strengthening the Reporting of Observational Studies in Epidemiology (STROBE) reporting guideline.^16^

### Participants and Data Sources

**Figure 1.**
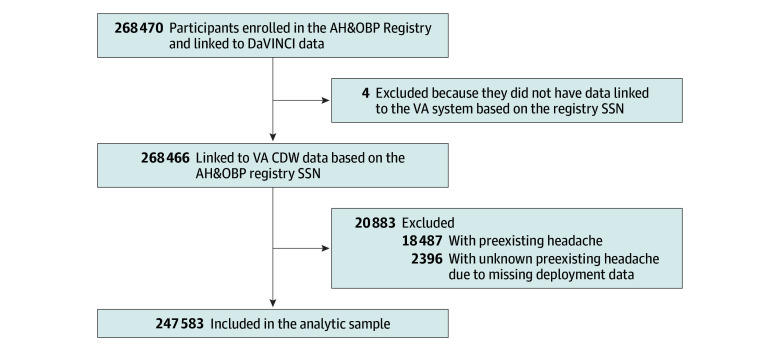
Flowchart of Airborne Hazards and Open Burn Pit (AH&OBP) Registry Participants in the Analytic Sample (April 1, 2014, to October 31, 2022) DaVINCI indicates US Department of Defense and US Department of Veterans Affairs Informatics and Computing Infrastructure; SSN, Social Security number; VA, Department of Veterans Affairs; VA CDW, VA Corporate Data Warehouse.

To describe the association between participants’ self-reported open burn pit exposure and headache, data from one registry (ie, AH&OBP Registry) and 2 separate health care systems (ie, DOD and VA) EHRs were linked. Beginning with data from the 268 470 current and former service members who participated in the AH&OBP Registry from April 1, 2014, through October 31, 2022, registry data were linked to VA Corporate Data Warehouse data via Social Security numbers. Data from 4 participants (0.0015% of participants) did not link to the VA system and were removed from analyses. Next, and noting at this time that DOD and VA health care systems have separate EHRs, data from the remaining participants were then linked to DOD and VA Informatics and Computing Infrastructure (DaVINCI) data (ie, DOD data). After excluding those with preexisting headache, the final analytic sample included data from 247 583 participants (Figure 1).

### Exposure and Outcome Definitions

Two open burn pit exposure composite variables based on the registry questionnaire were examined, including (1) a 5-level categorical variable (ie, being near an open burn pit and having duties associated with open burn pits, being near an open burn pit and open burn pit duties unknown, being near an open burn pit without having open burn pit duties, not being near an open burn pit nor having duties associated with open burn pit [ie, no exposure group], and missing) and (2) cumulative exposure gathered across deployments (a 6-level categorical variable: 0 days [ie, no exposure group], 1-184 days, 185-289 days, 290-448 days, >448 days, and missing). Open burn pit exposure was determined by positive responses from the AH&OBP Registry regarding proximity to open burn pits and open burn pit-related duties (eg, trash hauling and open burning). These were binary variables by deployment segment. Deployment segments in the AH&OBP Registry were generated by the DOD, which verified all deployment segments.^6^ However, 26.9% of deployment segments were missing exposure data; therefore, the Headache Centers of Excellence data analysis team created a no exposure group of those who indicated they were not near the open burn pit during any of their deployment segments. Participants who did not provide open burn pit exposure information on 1 or more of their deployment segments were not included in the no exposure group. To keep all participants in the analysis, those who did not provide information on whether they were near an open burn pit on any of their deployment segments were included as a missing group. Those who indicated they were near an open burn pit during at least 1 of their deployment segments were considered to have had open burn pit exposure. A variable was similarly defined for open burn pit duties exposure. Participants who indicated they had open burn pit duties during any of their deployment segments were considered to have open burn pit duties exposure. Those who specified that they did not have open burn pit duties exposure during any of their deployment segments did not have any exposure to open burn pit duties. Again, to be conservative, this did not include individuals with unknown open burn pit duty status for 1 or more of their deployment segments. Additional details regarding the methods, including the data element definitions, are included in eAppendix 4 in Supplement 1.

To examine the primary outcomes of incident headache and migraine, registry participants with preexisting headache (ie, headache coded before the date of first open burn pit exposure for those who were exposed or before the date of first deployment for those not exposed) were removed from the sample. Two primary outcomes included any medically diagnosed headache disorder or migraine obtained from *International Classification of Diseases, Ninth Revision, Clinical Modification* (*ICD-9-CM*) and *International Statistical Classification of Diseases and Related Health Problems, Tenth Revision* (*ICD-10*) codes from the EHR within the VHA (fiscal years 2008-2022) Headache Cohort or DOD DaVINCI datasets. Medically diagnosed is defined as headache diagnoses assigned by a health care practitioner via an *ICD* code and documented in the DOD and/or VA EHR (eAppendix 3 in Supplement 1). A secondary outcome included self-reported migraine that resulted in functional limitations (ie, disabling migraine).

### Statistical Analysis

The analysis was conducted between November 1, 2022, and January 31, 2024. Numbers (percentages) described categorical variables, whereas means (SDs) and medians (IQRs) described continuous variables. Separate multivariable logistic regression models determined the association of open burn pit status with each headache outcome, adjusting for confounding effects at baseline, such as age, sex, race and ethnicity, branch of military service, and presence of traumatic brain injury obtained via EHR data. Medically diagnosed headache was inclusive of migraine, and those with migraine had both headache outcomes. For each headache outcome, open burn pit exposure was modeled separately using the 5-level categorical exposure variable and then using the cumulative exposure quartiles. Cumulative exposure models were additionally adjusted for open burn pit duties exposure. Data were examined for missing values and violations of assumptions, with the latter including visual methods. All analyses considered 2-tailed *P* < .05 as statistically significant. All analyses were conducted using SAS software, version 9.4 (SAS Institute Inc).

### Sensitivity Analyses

Several sensitivity analyses were also conducted on medically diagnosed headache and migraine to understand the robustness of our data and included^17^ (1) retaining veterans with preexisting headache and statistical adjustment for preexisting headache status; (2) open burn pit exposure and open burn pit duties as separate covariates in models without an interaction term; (3) modeling headache outcomes where missing exposure information was not available; and (4) a multinomial logistic regression model to examine the effects of the 5-level categorical exposure variable on a combined headache outcome, comparing those with migraine, nonmigraine headache disorders, and no headache disorders. Veterans who had both migraine and a nonmigraine headache disorder were considered to have migraine (eTables 1-8 in Supplement 1).

## Results

### Characteristics of Participants in the Final Analytic Sample

The analytic sample included 247 583 veterans (222 498 [89.9%] male and 25 085 [10.1%] female) who were relatively young (mean [SD] age, 27.9 [7.7] years), with 24 460 (9.9%) Hispanic veterans, 31 334 (12.7%) non-Hispanic African American veterans, 149 676 (60.5%) non-Hispanic White veterans, 29 102 (11.8%) of unknown race and ethnicity, and 13 011 (5.3%) of other race or ethnicity (ie, Alaska Native or American Indian, Asian, Native Hawaiian or Other Pacific Islander, and multirace) (Table). A total of 150 842 veterans (60.9%) served in the US Army and 125 862 (50.8%) were medically diagnosed with at least 1 headache, with 61 812 (25.0%) having migraine (Table). Veterans with the highest amount of open burn pit exposure (ie, being near open burn pit and having duties associated with open burn pit exposure; n = 139 658 [56.4%]) were the largest group represented in the sample and were younger (mean [SD] age, 26.4 [6.9] years), had a smaller percentage of women veterans (11 343 [8.1%]), were more likely to be of Hispanic or African American race and ethnicity (15 946 [11.4%] and 20 487 [14.7%], respectively), and served in the US Army (95 409 [68.3%]). Compared with those without open burn pit exposure, those with the greatest amount of open burn pit exposure had the highest rates of developing any type of headache (77 903 [55.8%] vs 906 [40.4%]), including migraine (40 046 [28.7%] vs 408 [18.2%]).

**Table.** Characteristics of Veterans by Open Burn Pit Exposure Group^a^

Characteristic^b^	Near open burn pit and had open burn pit duties (n = 139 658)	Near open burn pit but unknown open burn pit duties (n = 38 774)	Near open burn pit but no open burn pit duties (n = 45 299)	Not near open burn pit and no open burn pit duties (n = 2241)	Missing (n = 21 611)	Total (N = 247 583)
Age at baseline, mean (SD), y	26.4 (6.8)	30.0 (8.2)	30.4 (8.5)	28.8 (8.1)	28.3 (7.9)	27.9 (7.7)
Sex						
Male	128 315 (91.9)	34 245 (88.3)	39 589 (87.4)	1948 (86.9)	18 401 (85.1)	222 498 (89.9)
Female	11 343 (8.1)	4529 (11.7)	5710 (12.6)	293 (13.1)	3210 (14.9)	25 085 (10.1)
Race and ethnicity						
Hispanic	15 946 (11.4)	2917 (7.5)	3563 (7.9)	171 (7.6)	1863 (8.6)	24 460 (9.9)
Non-Hispanic African American	20 487 (14.7)	3400 (8.8)	4617 (10.2)	253 (11.3)	2577 (11.9)	31 334 (12.7)
Non-Hispanic White	80 779 (57.8)	25 293 (65.2)	29 103 (64.2)	1415 (63.4)	13 086 (60.6)	149 676 (60.5)
Unknown	14 765 (10.6)	5185 (13.4)	5953 (13.1)	285 (12.7)	2914 (13.5)	29 102 (11.8)
Other^c^	7681 (5.5)	1979 (5.1)	2063 (4.6)	117 (5.2)	1171 (5.2)	13 011 (5.3)
Branch of US military service						
Army	95 409 (68.3)	21 484 (55.4)	22 802 (50.3)	973 (43.4)	10 174 (47.1)	150 842 (60.9)
Marine Corps	20 766 (14.9)	2662 (6.9)	4015 (8.9)	176 (7.8)	1698 (7.9)	29 317 (11.8)
Air Force	16 151 (11.6)	11 393 (29.4)	13 377 (29.5)	526 (23.5)	6886 (31.9)	48 333 (19.5)
Navy	6986 (5.0)	3129 (8.1)	4848 (10.7)	530 (23.6)	2691 (12.4)	18 184 (7.3)
Coast Guard	131 (0.1)	72 (0.2)	172 (0.4)	26 (1.2)	107 (0.5)	508 (0.2)
Unknown	215 (0.2)	34 (0.1)	85 (0.2)	10 (0.4)	55 (0.2)	399 (0.2)
TBI^d^						
Negative screen result	66 161 (47.4)	22 259 (57.4)	24 882 (54.9)	1032 (46.0)	11 376 (52.6)	125 710 (50.8)
Not screened	42 435 (30.4)	12 110 (31.2)	15 726 (34.7)	1083 (48.3)	8325 (38.5)	79 679 (32.2)
Positive screen result	31 062 (22.2)	4405 (11.4)	4691 (10.4)	126 (5.6)	1910 (8.8)	42 194 (17.0)
Medically diagnosed with headache at DOD or VA^e^						
Any headache	77 903 (55.8)	17 392 (44.8)	20 022 (44.2)	906 (40.4)	9639 (44.6)	125 862 (50.8)
Migraine headache	40 046 (28.7)	7757 (20.0)	9122 (20.1)	408 (18.2)	4479 (20.7)	61 812 (25.0)
Postwhiplash headache	10 826 (7.8)	2525 (6.5)	2800 (6.2)	119 (5.3)	1333 (6.2)	17 603 (7.1)
Posttraumatic headache	7348 (5.3)	987 (2.6)	1027 (2.3)	34 (1.5)	415 (1.9)	9811 (4.0)
Cluster headache	1363 (1.0)	306 (0.8)	351 (0.8)	15 (0.7)	163 (0.8)	2198 (0.9)

Abbreviations: DOD, US Department of Defense; TBI, traumatic brain injury; VA, US Department of Veterans Affairs.

^a^ Data are presented as number (percentage) of participants unless otherwise indicated.

^b^ DOD and VA Informatics and Computing Infrastructure data and VA Corporate Data Warehouse data were used to extract age, gender, race and ethnicity, and branch of service.

^c^ This group includes Alaska Native or American Indian, Asian, Native Hawaiian or Other Pacific Islander, and multirace.

^d^ The TBI screen was from the VA Health Factors section in the VA Corporate Data Warehouse data.

^e^ Medical practitioners assigned medically diagnosed headaches using *International Classification of Diseases, Ninth Revision, Clinical Modification* and *International Statistical Classification of Diseases and Related Health Problems, Tenth Revision* codes.

### Risk of Medically Diagnosed Headache and Migraine Among Open Burn Pit Exposure Group 

#### Any Headache Disorder

**Figure 2.**
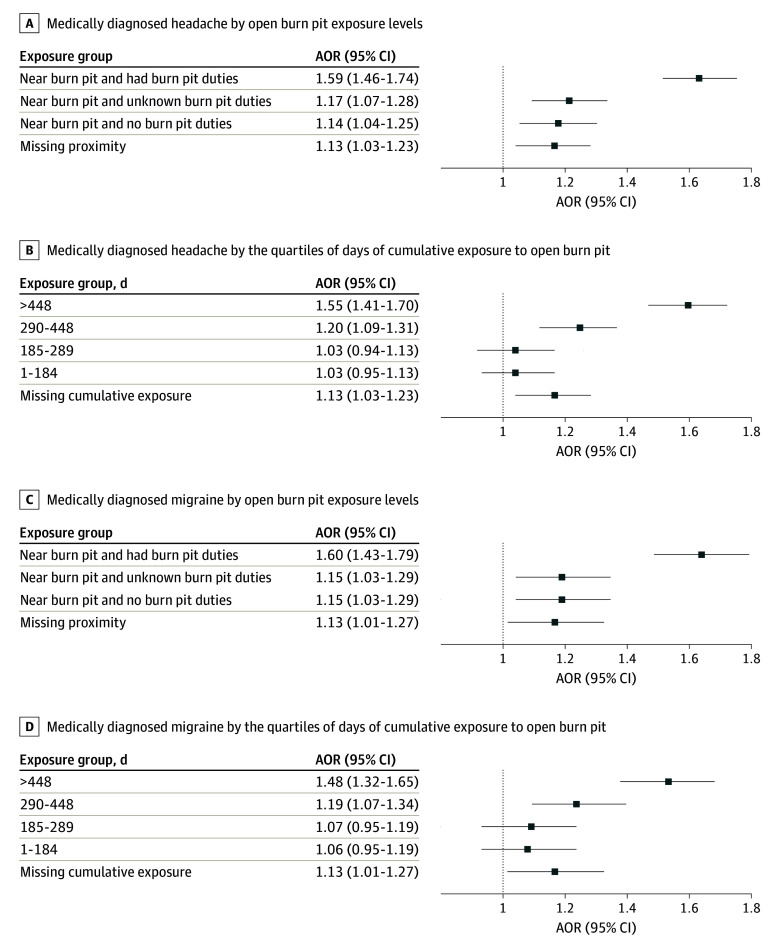
Adjusted Odds Ratios (AORs) for Medically Diagnosed Headache and Migraine by Amount of Open Burn Pit Exposure Compared With No Burn Pit Exposure Diagnoses of medically diagnosed headache and migraine were assigned by medical practitioners accordingly in clinical encounters (*International Classification of Diseases, Ninth Revision, Clinical Modification* and *International Statistical Classification of Diseases and Related Health Problems, Tenth Revision* diagnostic codes). No open burn pit exposure was the reference group for panels A and C, and 0 days of cumulative exposure served as the reference group for panels B and D. Odds ratios were adjusted for age, sex, race and ethnicity, branch of service, and presence of traumatic brain injury. Error bars indicate 95% CIs.

Participants in the highest open burn pit exposure group had significantly higher adjusted odds (adjusted odds ratio [AOR], 1.59; 95% CI, 1.46-1.74) of medically diagnosed headache (Figure 2A) compared with participants without exposure to open burn pits (ie, not being near an open burn pit and having no duties associated with an open burn pit). Those participants who reported being near open burn pits without open burn pit duties had an AOR of 1.14 (95% CI, 1.04-1.25) of being medically diagnosed with headache compared with participants without exposure. In a model that adjusted for a status of open burn pit duties, only the highest 2 quartiles of cumulative burn pit exposure (290-448 days and >448 days) had significantly higher adjusted odds of headache than those without exposure (290-448 days: AOR, 1.20; 95% CI, 1.09-1.31; >448 days: AOR, 1.55; 95% CI, 1.41-1.70) and migraine (290-448 days: AOR, 1.19; 95% CI, 1.07-1.34; >448 days: AOR, 1.48; 95% CI, 1.32-1.65) (Figure 2B). Increasing adjusted odds of medically diagnosed headache with increasing cumulative exposure starting at 290 days were observed.

#### Migraine

Participants who were near an open burn pit and had open burn pit duties had the highest adjusted odds (AOR, 1.60; 95% CI, 1.43-1.79) of medically diagnosed migraine (Figure 2C) compared with participants without exposure to open burn pits. Those who were near an open burn pit without open burn pit duties had increased adjusted odds (AOR, 1.15; 95% CI, 1.03-1.29) of medically diagnosed migraine compared with participants without exposure (Figure 2C). Adjusting for open burn pit duties, only the highest 2 quartiles of cumulative exposure (290-448 days and >448 days) had significantly higher adjusted odds of migraine than those without exposure (Figure 2D). Increasing odds of migraine with increasing cumulative exposure starting at 290 days were observed.

### Risk of Self-Reported Disabling Migraine Among Burn Pit Exposure Groups

Participants who were near an open burn pit with open burn pit duties had the highest adjusted odds (AOR, 1.93; 95% CI, 1.69-2.20) of self-reported disabling migraine compared with participants without exposure (eTable 3 in Supplement 1). Similarly, those near open burn pits and without open burn pit duties had significantly higher adjusted odds compared with those without exposure (AOR, 1.20; 95% CI, 1.05-1.37). Adjusting for open burn pit duties, all quartiles of cumulative exposure had significantly higher odds of disabling self-reported migraine headache compared with those without open burn pit exposure. However, increasing odds of migraine with increasing cumulative exposure were not observed (eTable 3 in Supplement 1). Sensitivity analyses did not change the associations between open burn pit exposure and medically diagnosed headache or migraine.

## Discussion

Airborne hazard exposure from the smoke and fumes of open burn pits that were routinely used by forward operating bases to incinerate waste materials may have short- and long-term health implications for those who served in Iraq, Afghanistan, and other areas of the Southwest Asia theater of military operations.^18^ This study is the first, to our knowledge, to report on associations between open burn pit exposure and incident headache, including migraine. A dose-dependent association exists between the amount (as indicated by cumulative number of days) of open burn pit exposure and being diagnosed with a new headache disorder by a health care practitioner, independent of sociodemographic and military characteristics, including presence of traumatic brain injury. A non–dose-dependent association exists between open burn pit exposure and developing self-reported severe, disabling migraine.

Service members and veterans have demonstrated an increased risk of headache and migraine attributable to specific aspects of being in the military apart from airborne hazard exposure. A large, population-based US military cohort study^19^ found that deployed personnel who reported combat exposure had significantly higher adjusted odds of any headache disorder (AOR, 1.72; 95% CI, 1.55-1.90), migraine (AOR, 1.53; 95% CI, 1.28-1.83), or severe headache (AOR, 1.79; 95% CI, 1.60-2.01) compared with those who were not deployed. While many headache triggers are common among active-duty service members and civilians, stress-related triggers were significantly more common among service members both with (73%) and without (69%) a history of head trauma compared to civilians (45%, *P* = .03).^20^ We report a high base rate of being diagnosed by a health care practitioner with incident headache disorder (40.4%) or migraine (18.2%) among those reported as not having exposure to open burn pits.

While studies examining airborne hazard and headache within the military community are lacking, associations between airborne hazard exposure and headache have been explored in nonmilitary settings. The WTC Health Registry conducted baseline interviews among survivors from 9/11 to understand the health impact of exposure to the airborne hazard produced by the combination of ignited jet fuel, including polycyclic aromatic hydrocarbons and volatile organic compounds, and particulate matter from the collapsed and damaged towers.^2^ In the few years after 9/11 among adult WTC survivors who were neither first responders nor clean-up workers, headache was one of the most common nonrespiratory conditions reported. Survivors exposed to dust and debris were more likely to report severe headaches compared with those who did not have the same airborne hazard exposure (26.2% vs 12.3%), after adjusting for age, race and ethnicity, sex, level of psychological distress, smoking status, and mode of recruitment (AOR, 2.0; 95% CI, 1.80-2.30).^2^ Another study^1^ using data from medical record review and data collection forms collected in the immediate aftermath of 9/11 noted that headache was reported among 46.8% of WTC rescue workers; headache rates were highest among construction workers (89.4%) and firefighters (72.4%).

### Limitations

The National Academies of Sciences, Engineering, and Medicine assessed the AH&OBP Registry and noted methodologic concerns related to observational epidemiologic studies, including selection bias and lack of a gold standard exposure-assessment tool by which to judge the magnitude and direction of self-reported exposure information.^9,15^ We strengthened this investigation through the inclusion of EHR data to aid in overcoming self-report bias with respect to headache outcome and the removal of preexisting headache to better examine the temporal sequence of exposure and outcome. We also conducted multiple sensitivity analyses, which demonstrated strength, consistency, and a biological gradient between burn pit exposure and headache disorders.^21^ Self-reported disabling migraine was specifically examined as a secondary outcome for comparison to past investigations of other health conditions that used only the registry questionnaire for health information. Because we modeled developing new-onset headache during the broad time window of April 1, 2014, through October 31, 2020, we could not address the time from exposure to developing headache within smaller units of time (eg, yearly). Next, there is no systematic environmental measure of toxic chemical exposure from military open burn pits. The toxin composition and amount of toxin entering the air are likely to vary across time, open burn pit sites, open burn pit size, and local weather conditions. Therefore, proxies for “dose” of open burn pit exposure were created by comparing persons with and without open burn pit duties among those near open burn pits and comparing persons with varying lengths of time being near burn pits across deployments. This heterogeneity also makes understanding the pathophysiologic underpinnings of our findings difficult; airborne hazards may access the central nervous system through the systemic circulation, respiratory tract, or olfactory tract, leading to oxidative stress, neuroinflammation, and trigeminovascular system activation.^22,23^ Our analyses also did not address specific subgroups of service personnel with anticipated higher airborne hazard exposures (eg, airfield or flight deck personnel, fuel motor transport operators, and others); further work should delve into multiple airborne hazards.

## Conclusions

Among service personnel who participated in the VA AH&OBP Registry who previously had no history of headache,^24^ rates of developing new headache were highest among those who reported being near open burn pits and having duties associated with open burn pit exposure, intermediate among those who reported being near open burn pits and not having duties associated with open burn pit exposure, and lowest among those with no exposure to open burn pits. After adjusting for important factors known to increase the risk of developing headache, including the presence of traumatic brain injury, the odds of any headache examined were highest among service members with the most exposure to open burn pits. These data indicate that the greater the amount of open burn pit exposure service members experience, the more likely they are to develop new-onset headache. These new data identify potentially important associations between open burn bit exposure and new-onset headache among service personnel as well as a possible health condition that may be encountered more frequently in VHA facilities in the context of mandatory screening for military exposures as part of the Honoring our PACT Act of 2022.

## Notes

Supplement 1. **eAppendix 1.** Public Law 112-260 (selected section) **eAppendix 2.** Airborne Hazards and Open Burn Pit Registry Data Dictionary **eAppendix 3.** Diagnostic Codes for Medically Diagnosed Headache & Migraine **eAppendix 4.** Description of Data Elements **eTable 1.** Overall Headache Including Pre-Existing Headache Diagnosed by Open Burn Pit Exposure Groups & Cumulative Exposure **eTable 2.** Migraine Including Pre-Existing by Open Burn Pit Exposure Groups & Cumulative Exposure **eTable 3.** Multinomial Logistic Regression Model Comparing Migraine and Other Headache Disorder with No Headache by Open Burn Pit Exposure Groups **eTable 4.** Overall Headache Diagnosed by Near Open Burn Pit Exposure and Open Burn Pit Duties **eTable 5.** Migraine Diagnosed by Near Open Burn Pit Exposure and Open Burn Pit Duties **eTable 6.** Overall Headache Diagnosed by Exposure Groups Excluding Those with Missing Open Burn Pit Exposure Information **eTable 7.** Migraine Diagnosed by Near Open Burn Pit Exposure and Open Burn Pit Duties Excluding Those with Missing Open Burn Pit Exposure Information **eTable 8.** Self-Reported Disabling Migraine by Open Burn Pit Exposure Groups

Supplement 2.
Data Sharing Statement
