# Supplementary material for: Open Burn Pit Exposure in Headache Disorder and Migraine
Source: JAMA Netw Open. 2024 Sep 4;7(9):e2431522. doi: 10.1001/jamanetworkopen.2024.31522 (PMC11375476; doi:10.1001/jamanetworkopen.2024.31522)
Supplement: Supplement 1. — eAppendix 1. Public Law 112-260 (selected section) eAppendix 2. Airborne Hazards and Open Burn Pit Registry Data Dictionary eAppendix 3. Diagnostic Codes for Medically Diagnosed Headache & Migraine eAppendix 4. Description of Data Elements eTable 1. Overall Headache Including Pre-Existing Headache Diagnosed by Open Burn Pit Exposure Groups & Cumulative Exposure eTable 2. Migraine Including Pre-Existing by Open Burn Pit Exposure Groups & Cumulative Exposure eTable 3. Multinomial Logistic Regression Model Comparing Migraine and Other Headache Disorder with No Headache by Open Burn Pit Exposure Groups eTable 4. Overall Headache Diagnosed by Near Open Burn Pit Exposure and Open Burn Pit Duties eTable 5. Migraine Diagnosed by Near Open Burn Pit Exposure and Open Burn Pit Duties eTable 6. Overall Headache Diagnosed by Exposure Groups Excluding Those with Missing Open Burn Pit Exposure Information eTable 7. Migraine Diagnosed by Near Open Burn Pit Exposure and Open Burn Pit Duties Excluding Those with Missing Open Burn Pit Exposure Information eTable 8. Self-Reported Disabling Migraine by Open Burn Pit Exposure Groups [file jamanetwopen-e2431522-s001.pdf]

Supplementary Online Content

Sico JJ, Anthony SE, Phadke M, et al. Open burn pit exposure in headache disorder and migraine. *JAMA Netw Open*. 2024;7(9):e2431522. doi:10.1001/jamanetworkopen.2024.31522

eAppendix 1. Public Law 112-260 (selected section)

eAppendix 2. Airborne Hazards and Open Burn Pit Registry Data Dictionary

eAppendix 3. Diagnostic Codes for Medically Diagnosed Headache & Migraine

eAppendix 4. Description of Data Elements

eTable 1. Overall Headache Including Pre-Existing Headache Diagnosed by Open Burn Pit Exposure Groups & Cumulative Exposure

eTable 2. Migraine Including Pre-Existing by Open Burn Pit Exposure Groups & Cumulative Exposure

eTable 3. Multinomial Logistic Regression Model Comparing Migraine and Other Headache Disorder with No Headache by Open Burn Pit Exposure Groups

eTable 4. Overall Headache Diagnosed by Near Open Burn Pit Exposure and Open Burn Pit Duties

eTable 5. Migraine Diagnosed by Near Open Burn Pit Exposure and Open Burn Pit Duties

eTable 6. Overall Headache Diagnosed by Exposure Groups Excluding Those with Missing Open Burn Pit Exposure Information

eTable 7. Migraine Diagnosed by Near Open Burn Pit Exposure and Open Burn Pit Duties Excluding Those with Missing Open Burn Pit Exposure Information

eTable 8. Self-Reported Disabling Migraine by Open Burn Pit Exposure Groups

This supplementary material has been provided by the authors to give readers additional information about their work.

© 2024 Sico JJ et al. *JAMA Network Open.*

Appendix 1. Public Law 112-260 (selected section)

Public Law 112–260
112th Congress

An Act

To amend title 38, United States Code, to ensure that deceased veterans with no known next of kin can receive a dignified burial, and for other purposes.

Jan. 10, 2013
[S. 3202]

Be it enacted by the Senate and House of Representatives of the United States of America in Congress assembled,

Dignified Burial and Other Veterans' Benefits Improvement Act of 2012.
38 USC 101 note.

SECTION 1. SHORT TITLE; TABLE OF CONTENTS.

(a) SHORT TITLE.—This Act may be cited as the "Dignified Burial and Other Veterans' Benefits Improvement Act of 2012".

(b) TABLE OF CONTENTS.—The table of contents for this Act is as follows:

Sec. 1. Short title; table of contents.
Sec. 2. Scoring of budgetary effects.

TITLE I—CEMETERY MATTERS

Sec. 101. Furnishing caskets and urns for deceased veterans with no known next of kin.
Sec. 102. Veterans freedom of conscience protection.
Sec. 103. Improved communication between Department of Veterans Affairs and medical examiners and funeral directors.
Sec. 104. Identification and burial of unclaimed or abandoned human remains.
Sec. 105. Exclusion of persons convicted of committing certain sex offenses from interment or memorialization in national cemeteries, Arlington National Cemetery, and certain State veterans' cemeteries and from receiving certain funeral honors.
Sec. 106. Restoration, operation, and maintenance of Clark Veterans Cemetery by American Battle Monuments Commission.
Sec. 107. Report on compliance of Department of Veterans Affairs with industry standards for caskets and urns.

TITLE II—HEALTH CARE

Sec. 201. Establishment of open burn pit registry.
Sec. 202. Transportation of beneficiaries to and from facilities of Department of Veterans Affairs.
Sec. 203. Extension of reduced pension for certain veterans covered by medicaid plans for services furnished by nursing facilities.
Sec. 204. Extension of report requirement for Special Committee on Post-Traumatic-Stress Disorder.

TITLE III—OTHER MATTERS

Sec. 301. Off-base transition training for veterans and their spouses.
Sec. 302. Requirement that judges on United States Court of Appeals for Veterans Claims reside within 50 miles of District of Columbia.
Sec. 303. Designation of Trinka Davis Veterans Village.
Sec. 304. Designation of William "Bill" Kling Department of Veterans Affairs Outpatient Clinic.
Sec. 305. Designation of Mann-Grandstaff Department of Veterans Affairs Medical Center.
Sec. 306. Designation of David F. Winder Department of Veterans Affairs Community Based Outpatient Clinic.

© 2024 Sico JJ et al. *JAMA Network Open.*

TITLE II—HEALTH CARE

38 USC 527 note. **SEC. 201. ESTABLISHMENT OF OPEN BURN PIT REGISTRY.**

(a) ESTABLISHMENT OF REGISTRY.—

Deadline. (1) IN GENERAL.—Not later than one year after the date of the enactment of this Act, the Secretary of Veterans Affairs shall—

(A) establish and maintain an open burn pit registry for eligible individuals who may have been exposed to toxic airborne chemicals and fumes caused by open burn pits;

(B) include any information in such registry that the Secretary of Veterans Affairs determines necessary to ascertain and monitor the health effects of the exposure of members of the Armed Forces to toxic airborne chemicals and fumes caused by open burn pits;

(C) develop a public information campaign to inform eligible individuals about the open burn pit registry, including how to register and the benefits of registering; and

Notification. (D) periodically notify eligible individuals of significant developments in the study and treatment of conditions associated with exposure to toxic airborne chemicals and fumes caused by open burn pits.

(2) COORDINATION.—The Secretary of Veterans Affairs shall coordinate with the Secretary of Defense in carrying out paragraph (1).

(b) REPORT TO CONGRESS.—

Contracts. (1) REPORTS BY INDEPENDENT SCIENTIFIC ORGANIZATION.—The Secretary of Veterans Affairs shall enter into an agreement with an independent scientific organization to prepare reports as follows:

(A) Not later than two years after the date on which the registry under subsection (a) is established, an initial report containing the following:

Assessment. (i) An assessment of the effectiveness of actions taken by the Secretaries to collect and maintain information on the health effects of exposure to toxic

© 2024 [illegible] et al. JAMA Network Open.

airborne chemicals and fumes caused by open burn pits.

(ii) Recommendations to improve the collection and maintenance of such information. Recommendations.

(iii) Using established and previously published epidemiological studies, recommendations regarding the most effective and prudent means of addressing the medical needs of eligible individuals with respect to conditions that are likely to result from exposure to open burn pits.

(B) Not later than five years after completing the initial report described in subparagraph (A), a follow-up report containing the following:

(i) An update to the initial report described in subparagraph (A).

(ii) An assessment of whether and to what degree the content of the registry established under subsection (a) is current and scientifically up-to-date. Assessment.

(2) SUBMITTAL TO CONGRESS.—

(A) INITIAL REPORT.—Not later than two years after the date on which the registry under subsection (a) is established, the Secretary of Veterans Affairs shall submit to Congress the initial report prepared under paragraph (1)(A).

(B) FOLLOW-UP REPORT.—Not later than five years after submitting the report under subparagraph (A), the Secretary of Veterans Affairs shall submit to Congress the follow-up report prepared under paragraph (1)(B).

(c) DEFINITIONS.—In this section:

(1) ELIGIBLE INDIVIDUAL.—The term “eligible individual” means any individual who, on or after September 11, 2001—

(A) was deployed in support of a contingency operation while serving in the Armed Forces; and

(B) during such deployment, was based or stationed at a location where an open burn pit was used.

(2) OPEN BURN PIT.—The term “open burn pit” means an area of land located in Afghanistan or Iraq that—

(A) is designated by the Secretary of Defense to be used for disposing solid waste by burning in the outdoor air; and

(B) does not contain a commercially manufactured incinerator or other equipment specifically designed and manufactured for the burning of solid waste.

LEGISLATIVE HISTORY—S. 3202:

© 2024 Sico JJ et al. *JAMA Network Open.*

CONGRESSIONAL RECORD, Vol. 158 (2012):

Dec. 19, considered and passed Senate. Dec. 30, considered and passed House.

Appendix 2: Airborne Hazards and Open Burn Pit Registry Data Dictionary

1.) Registry Identifier Variables

Variable Name	Description	Format
ahobpr_registrant_id	Unique registrant identifier, used for merging AHOBPR datasets	
ssn	Registrant Social Security Number	
birth_date	Registrant Date of Birth	MMDDYY10.
first_name	Registrant First Name	
middle_name	Registrant Middle Name	
last_name	Registrant Last Name	

2.) Registry Questionnaire Variables

Identifier Type	Variable_Name	Description	Format	Category
General	Questionnaire_CompletedDate	Date and time questionnaire submitted	DATE9.	
General	AgeRegister	Age in years at questionnaire completion		
General	ServiceBranch	Service Branch at AHOBPR registration	$BRANCH.	A = Army<br>F = Air Force<br>N = Navy<br>M = Marine Corps<br>C = Coast Guard<br>P = Public Health Service<br>U=Unknown
General	deceased_flag	deceased_flag		1 = Deceased
Symptoms and Medical History	DifficultyJog	2.1.A - How difficult is it to run or jog one mile on a level surface?	FUNCDIFF.	0 = Not at all difficult<br>1 = Only a little difficult<br>2 = Somewhat difficult<br>3 = Very difficult<br>4 = Can't do it at all<br>5 = Do not do this activity<br>8888 = I do not wish to answer<br>9999 = Don't know

Symptoms and Medical History	DifficultyWalk	2.1.B - How difficult is it to walk on a level surface for one mile?	FUNCDIFF.	0 = Not at all difficult<br>1 = Only a little difficult<br>2 = Somewhat difficult<br>3 = Very difficult<br>4 = Can't do it at all<br>5 = Do not do this activity<br>8888 = I do not wish to answer<br>9999 = Don't know
Symptoms and Medical History	DifficultyShortWalk	2.1.C - How difficult is it to walk a 1/4 of a mile - about 3 city blocks?	FUNCDIFF.	0 = Not at all difficult<br>1 = Only a little difficult<br>2 = Somewhat difficult<br>3 = Very difficult<br>4 = Can't do it at all<br>5 = Do not do this activity<br>8888 = I do not wish to answer<br>9999 = Don't know
Symptoms and Medical History	DifficultyHill	2.1.D - How difficult is it to walk up a hill or incline?	FUNCDIFF.	0 = Not at all difficult<br>1 = Only a little difficult<br>2 = Somewhat difficult<br>3 = Very difficult<br>4 = Can't do it at all<br>5 = Do not do this activity<br>8888 = I do not wish to answer<br>9999 = Don't know

© 2024 Sico JJ et al. *JAMA Network Open.*

Symptoms and Medical History	DifficultyStairs	2.1.E - How difficult is it to walk up 10 steps or climb a flight of stairs?	FUNCDIFF.	0 = Not at all difficult<br>1 = Only a little difficult<br>2 = Somewhat difficult<br>3 = Very difficult<br>4 = Can't do it at all<br>5 = Do not do this activity<br>8888 = I do not wish to answer<br>9999 = Don't know
Symptoms and Medical History	DifficultyMigraine	2.1.F - What condition or health problem causes you to have difficulty with these activities? Check all that apply. Migraine headaches (not just headaches)	YN.	1=Yes
Symptoms and Medical History	Allergies	2.2.1.A - Have you ever been told by a doctor or other health professional that you had Hay fever or allergies to pollen, dust, or animals?	YN.	1 = Yes<br>0 = No<br>8888 = I do not wish to answer<br>9999 = Don't Know
Symptoms and Medical History	Asthma	2.2.1.B - Have you ever been told by a doctor or other health care professional that you had asthma?	YN.	1 = Yes<br>0 = No<br>8888 = I do not wish to answer<br>9999 = Don't Know
Symptoms and Medical History	Emphysema	2.2.1.C - Have you ever been told by a doctor or other health care professional that you had emphysema?	YN.	1 = Yes<br>0 = No<br>8888 = I do not wish to answer<br>9999 = Don't Know
Symptoms and Medical History	ChronicBronchitis	2.2.1.D - Have you ever been told by a doctor or other health care professional that you had chronic bronchitis?	YN.	1 = Yes<br>0 = No<br>8888 = I do not wish to answer<br>9999 = Don't Know
Symptoms and Medical History	COPD	2.2.1.E - Have you ever been told by a doctor or other health care professional that you had chronic obstructive	YN.	1 = Yes<br>0 = No<br>8888 = I do not wish to answer<br>9999 = Don't Know

		pulmonary disease also called COPD?		
Symptoms and Medical History	OtherLung	2.2.1.F - Have you ever been told by a doctor or other health care professional that you had some lung disease or condition other than asthma, emphysema, chronic bronchitis or COPD?	YN.	1 = Yes<br>0 = No<br>8888 = I do not wish to answer<br>9999 = Don't Know
Symptoms and Medical History	ConstBronch	2.2.1.G - Have you ever been told by a doctor or other health care professional that you had constrictive bronchiolitis (CB)?	YN.	1 = Yes<br>0 = No<br>8888 = I do not wish to answer<br>9999 = Don't Know
Symptoms and Medical History	IPF	2.2.1.H - Have you ever been told by a doctor or other health care professional that you had idiopathic pulmonary fibrosis (IPF)?	YN.	1 = Yes<br>0 = No<br>8888 = I do not wish to answer<br>9999 = Don't Know
Symptoms and Medical History	LungDxBeforeDeploy	2.2.1.I - When you were told you had asthma, emphysema, chronic bronchitis, COPD, or some other lung disease by a doctor or other health care professional, were you told before, during, or after deployment? Before deployment	YN.	1=Yes<br>7777 = Not asked/Not applicable
Symptoms and Medical History	LungDXDuringDeploy	2.2.1.I - When you were told you had asthma, emphysema, chronic bronchitis, COPD, or some other lung disease by a doctor or other health care professional, were you told before, during, or after deployment? During deployment	YN.	1=Yes<br>7777 = Not asked/Not applicable
Symptoms and Medical History	LungDxAfterDeploy	2.2.1.I - When you were told you had asthma, emphysema, chronic bronchitis,	YN.	1=Yes<br>7777 = Not asked/Not applicable

© 2024 Sico JJ et al. *JAMA Network Open.*

		COPD, or some other lung disease by a doctor or other health care professional, were you told before, during, or after deployment? After deployment		
Symptoms and Medical History	LungDxNoAnswer	2.2.1.I - When you were told you had asthma, emphysema, chronic bronchitis, COPD, or some other lung disease by a doctor or other health care professional, were you told before, during, or after deployment? I do not wish to answer	YN.	1=Yes<br>7777 = Not asked/Not applicable
Symptoms and Medical History	LungDxDK	2.2.1.I - When you were told you had asthma, emphysema, chronic bronchitis, COPD, or some other lung disease by a doctor or other health care professional, were you told before, during, or after deployment? Don't know	YN.	1=Yes<br>7777 = Not asked/Not applicable
Symptoms and Medical History	LungDxChangeDeploy	2.2.1.J - Did this lung disease get better, worse, or about the same during deployment?	CHANGE.	0 = Better<br>1 = Worse<br>2 = About the same<br>7777 = Not applicable<br>8888 = I do not wish to answer<br>9999 = Don't know
Symptoms and Medical History	HTN	2.2.2.A - Have you ever been told by a doctor or other health care professional that you had hypertension, also called high blood pressure?	YN.	1 = Yes<br>0 = No<br>8888 = I do not wish to answer<br>9999 = Don't Know
Symptoms and Medical History	CAD	2.2.2.B - Have you ever been told by a doctor or other health care professional that you had coronary artery disease?	YN.	1 = Yes<br>0 = No<br>8888 = I do not wish to answer<br>9999 = Don't Know

© 2024 Sico JJ et al. *JAMA Network Open.*

Symptoms and Medical History	Angina	2.2.2.C - Have you ever been told by a doctor or other health care professional that you had angina pectoris?	YN.	1 = Yes<br>0 = No<br>8888 = I do not wish to answer<br>9999 = Don't Know
Symptoms and Medical History	MI	2.2.2.D - Have you ever been told by a doctor or other health care professional that you had a heart attack, also called myocardial infarction?	YN.	1 = Yes<br>0 = No<br>8888 = I do not wish to answer<br>9999 = Don't Know
Symptoms and Medical History	OtherHeart	2.2.2.E - Have you ever been told by a doctor or other health care professional that you had a heart condition other than coronary artery disease or angina or myocardial infarction?	YN.	1 = Yes<br>0 = No<br>8888 = I do not wish to answer<br>9999 = Don't Know
Symptoms and Medical History	HeartDxBeforeDeploy	2.2.2.F - When you were told you had hypertension, coronary artery disease, angina pectoris, a heart attack, or some other heart condition by a doctor or other health care professional, were you told before, during, or after deployment? (check all that apply) Before deployment	YN.	1=Yes<br>7777 = Not asked/Not applicable
Symptoms and Medical History	HeartDxDuringDeploy	2.2.2.F - When you were told you had hypertension, coronary artery disease, angina pectoris, a heart attack, or some other heart condition by a doctor or other health care professional, were you told before, during, or after deployment? (check all that apply) During deployment	YN.	1=Yes<br>7777 = Not asked/Not applicable

© 2024 Sico JJ et al. *JAMA Network Open.*

Symptoms and Medical History	HeartDxAfterDeploy	2.2.2.F - When you were told you had hypertension, coronary artery disease, angina pectoris, a heart attack, or some other heart condition by a doctor or other health care professional, were you told before, during, or after deployment? (check all that apply) After deployment	YN.	1=Yes<br>7777 = Not asked/Not applicable
Symptoms and Medical History	HeartDxNoAnswer	2.2.2.F - When you were told you had hypertension, coronary artery disease, angina pectoris, a heart attack, or some other heart condition by a doctor or other health care professional, were you told before, during, or after deployment? (check all that apply) I do not wish to answer	YN.	1=Yes<br>7777 = Not asked/Not applicable
Symptoms and Medical History	HeartDxDK	2.2.2.F - When you were told you had hypertension, coronary artery disease, angina pectoris, a heart attack, or some other heart condition by a doctor or other health care professional, were you told before, during, or after deployment? (check all that apply) Don't know	YN.	1=Yes<br>7777 = Not asked/Not applicable
Tobacco Exposure	Smoker	Derived Flag for Current Smoker	YN.	1 = Yes<br>0 = No
Tobacco Exposure	SmokeAge	2.5.B - How old were you when you first started to smoke fairly regularly?	SMOKEAGE.	1 - current age (years)<br>6666 = Never smoked regularly<br>7777 = Not asked/Not applicable

© 2024 Sico JJ et al. *JAMA Network Open.*

				8888 = I do not wish to answer<br>9999 = Don't know
Tobacco Exposure	CurrentCigFreq	2.5.C - Do you now smoke cigarettes every day, some days or not at all?	CURRENTCIGFREQ.	0 = Not at all<br>1 = Some days<br>2 = Every day<br>7777 = Not asked/Not applicable<br>8888 = I do not wish to answer<br>9999 = Don't know

3.) Registry Deployment Variables

**Identifier Type**	**Variable Name**	**Description**	**Format**	**Category**
Deployment	ahobpr_registrant_id	Unique registrant identifier, used for merging AHOBPR datasets		
Deployment	DeploymentStart	Start date of this deployment segment	MMDDYY10.	
Deployment	DeploymentEnd	End date of this deployment segment	MMDDYY10.	
Deployment	DeploymentDays	Duration of this deployment segment (days)		
Location Specific Deployment Exposures	BurnPit	Q1.2.D - Were you near a burn pit during these dates (on the base or close enough to the base for you to see the smoke)?	YN.	Yes<br>No<br>I do not wish to answer<br>Don't Know
Location Specific Deployment Exposures	BurnPitDuties	Q1.2.F - Did your duties during these dates include the burn pit (examples include trash burning, hauling trash to the burn pit, burn pit security, trash sorting at the burn pit)?	YN.	Yes<br>No<br>I do not wish to answer

© 2024 Sico JJ et al. *JAMA Network Open.*

Deployment	cum_burnpit_exposure_days	Duration of cumulative exposure at burnpit site (days)		

© 2024 Sico JJ et al. *JAMA Network Open.*

Appendix 3: Diagnostic Codes for Medically Diagnosed Headache & Migraine

International Classification of Disease Versions 9th and 10th Revision Clinical Modification Codes

Description	**ICD-9-CM**	**ICD-10-CM**
**Headache, not otherwise specified (NOS)**	784.0	R51., R51.0, R51.9
**Migraine**	346.XX	G43.XXX, G43.BX
Migraine, w/o aura	346.1X	G43.0XX
Migraine, w/ aura	346.0X	G43.1XX
Chronic Migraine	346.7X	G43.7XX
**Tension Headache**	307.81, 339.1X	G44.2XX
**Trigeminal Autonomic Cephalgias (TACs)**	339.0X, 339.41	G44.0XX, G44.51
Cluster Headache	339.00, 339.01, 339.02	G44.00X, G44.01X, G44.02X
Hemicrania	339.03, 339.04, 339.41	G44.03X, G44.04X, G44.51
**Other Primary Headache**	339.42, 339.43, 339.44, 339.8X	G43.CX, G44.52, G44.53, G44.54, G44.59, G44.8X
**Post-Traumatic Headache**	339.2X	G44.3XX
**Post-Whiplash Headache**	847.0	S13.4XXX, S13.8XXX, S13.9XXX
**Secondary Headache**	339.3	G44.1, G44.4X
Vascular Headache		G44.1
Drug-Induced Headache	339.3	G44.4X

© 2024 Sico JJ et al. *JAMA Network Open.*

Appendix 4: Description of the Data Elements

Headache Outcomes

Medically Diagnosed Headache and Migraine

The two primary outcomes included 1) medically diagnosed headache and 2) medically diagnosed migraine (*ICD-9-CM* and *ICD-10-CM*) obtained from the electronic health records (EHR) within the Veterans Health Administration (VHA) (fiscal years 2008- 2022) headache cohort or the Department of Defense (DoD) Department of Veterans Affairs Infrastructure for Clinical Intelligence (DaVINCI) data sets. The primary outcomes did not include participants with pre-existing headache. A DoD headache diagnosis was considered pre-existing if the diagnosis date was prior to the date of first open burn pit exposure for those who were exposed or prior to date of first deployment for those with no exposure. If an individual had a VHA diagnosed headache disorder and/or a DoD diagnosed headache disorder without the presence of any pre-existing headache, they were considered to have headache diagnosed by a healthcare provider. The same procedure was used to identify those with migraine diagnosed by a healthcare provider. These primary outcomes were modeled separately.

Self-Reported Disabling Migraine Headache

The secondary outcome, disabling migraine headache, was determined by a positive response to the AH&OBP Registry question **2.1.F** based on the following questions: **2.1.A** running or jogging on a level surface for one mile **2.1.B**. walking on a level surface for one mile, **2.1.C**. walking 3 city blocks, **2.1.D**. walking up an incline, **2.1.E**. walking up 10 steps or a flight of a stairs. Self-reported disabling migraine headache was modeled independently of medically diagnosed headache and migraine.

Open Burn Pit Exposure

Two open burn pit exposure composite variables consisted of: 1) open burn pit exposure group (a five-level categorical variable comprised of 2 binary variables on "setting" - being 'near open burn pit' and 'open burn pit duties') and 2) cumulative exposure gathered across deployments (a six-level categorical variable).

Open Burn Pit Exposure Group.

The responses from the Airborne Hazards and Open Burn Pit Registry (AH&OBP) Self-Assessment Questionnaire from April 2014 through October 2022 included the pilot testing period. Open burn pit exposure was determined by positive responses to questions regarding proximity to open burn pits and open burn pit-related duties (trash hauling, sorting, and open burning). These were binary variables by deployment segment. Deployment segments in the AH&OBP Registry were generated by the DoD who verified all deployment segments.[6] However, 26.9% of deployment segments were missing exposure data, therefore the Headache Center of Excellence (HCoE) data analysis team created a no exposure group of those who indicated they were not near the open burn pit during any of their deployment segments. To be conservative, participants who did not provide open burn pit exposure information on one or more of their deployment segments were not included in the no-exposure group. The no-exposure group constituted 2,424 (0.9%) individuals. To keep all participants in the analysis, those who did not provide information on whether they were near an open burn pit at any of their deployment segments were included as a "missing" group. Those who indicated they were near an open burn pit during at least one of their deployment segments were considered to have had open burn pit exposure.

A variable was similarly defined for open burn pit duties exposure. Participants who indicated they had open burn pit duties during any of their deployment segments were considered to have open burn pit duties exposure. Those who specified that they did not have open burn pit duties exposure during any of their deployment segments did not have any exposure to open burn pit duties. Again, to be conservative, this did not include individuals with unknown open burn pit duty status for one or more of their deployment segments.

Using a combination of these two open burn pit exposure variables, a five-level overall exposure variable was defined as:

1) Not near an open burn pit and did not have any open burn pit duties (reference).
2) Near an open burn pit but did not have open burn pit duties.
3) Near an open burn pit and had open burn pit duties.

4) Near an open burn pit but unable to determine if they had open burn pit duties (unknown open burn pit duties).
5) Missing information of whether they were near a open burn pit. (This was kept as a category in the outcome variable to avoid dropping a large portion of the sample in analysis n=21,611)

Cumulative Open burn Pit Exposure.

The cumulative exposure variable provided in the analytic dataset by the Airborne Hazard and Open burn Pit Center of Excellence was based upon the duration of cumulative exposure 'near open burn pit' site (days) and did not include open burn pit duty hours. The HCoE data analysis team created a categorized version of the provided cumulative exposure variable with six levels: a 'no exposure' group, a 'missing' group, and the remaining exposure levels were split into quartiles. A total of 21,611 (8.7%) participants were missing this variable.

Participant Covariates

Age (continuous): The most frequently noted birth date in the VA Corporate Data Warehouse (CDW) data was used as the date of birth for any participants missing date of birth in the registry data. A total of 1,128 persons were missing birth date in the registry data and this was reduced to 2 persons after inserting values available from the VA CDW data.

Age was calculated independently for the open burn pit exposure and open burn pit no-exposure groups. For the open burn pit exposure group, age was determined using birth date compared to the date of first deployment with open burn pit exposure. For the no-exposure group, age was calculated using birth date compared to the date of first deployment. To be conservative, for those who did not have exposure data the first date of their potential exposure was selected meaning their first deployment segment. A total of 1,028 participants were missing age due to unknown first deployment date.

Sex: As sex was not provided in the registry data, it was obtained from VA CDW data, which historically was limited to male and female. The most frequently noted sex in VA CDW data was used for each person. If both sexes were noted the same number of times for an individual in the CDW data, sex was selected at random from the first observation. Sex was obtained for every registry participant. To note, it was unclear if participants were answering questions regarding gender classification or biological sex.

Race and Ethnicity: Race and ethnicity information were not provided in the registry data, so this was obtained from VA CDW data. The most frequently noted race in the CDW data was considered that person's race. Race could not be determined for 37,033 registry participants and when combined with ethnicity information, this left 31,854 with unknown race/ethnicity. Race and ethnicity variables were combined with the following levels: non-Hispanic White, non-Hispanic African American, Hispanic (any race), Other, and Unknown.

Branch of Service: Branch of service was provided in the registry data but for those missing or unknown branch of service, branch of service was determined separately using both the VA CDW data and DoD DaVINCI data. 3,400 were missing branch of service in the registry data, with an additional 937 listed as unknown. VA data reduced this number to 1,761 and DoD DaVINCI data further reduced this to 417 with an unknown branch of service. The most frequently noted branch of service was determined using both data sources. A determination was not made for those with multiple branches having the same frequency.

Traumatic Brain Injury (TBI): The presence of TBI that was defined as a positive TBI screen at any time in the VA Health Factors within CDW data.

Supplementary Tables

eTable 1. Overall Headache Including Pre-Existing Headache Diagnosed by Open Burn Pit Exposure Groups & Cumulative Exposure

**Open Burn Pit Exposure Groups**[a]	**Adjusted Odds Ratio (95% CI)**[c]	**p value**
Near Open Burn Pit and Open Burn Pit Duties	1.56 (1.44-1.70)	<.001
Near Open Burn Pit and Unknown Open Burn Pit Duties	1.14 (1.05-1.25)	0.002
Near Open Burn Pit and No Open Burn Pit Duties	1.13 (1.04-1.24)	0.004
Missing	1.11 (1.02-1.22)	0.02
**Days of Cumulative Exposure**[b]		
>448 days	1.53 (1.40-1.67)	<.001
290- 448 days	1.19 (1.09-1.30)	<.001
185- 289 days	1.03 (0.95-1.13)	0.46
1-184 days	1.03 (0.95-1.13)	0.45
Missing	1.11 (1.02-1.22)	0.02

[a]Model was based on exposure groups. Reference group was 'Not Near Open Burn Pit and No Open Burn Pit Duties'.
[b]Model was based on cumulative exposure variable from the registry. Reference group was 0 days of open burn pit exposure.
[c]Odds ratios adjusted for pre-existing headache, age, sex, race and ethnicity, branch of service, and presence of traumatic brain injury.

© 2024 Sico JJ et al. *JAMA Network Open.*

eTable 2. Migraine Including Pre-Existing by Open Burn Pit Exposure Groups & Cumulative Exposure

**Open Burn Pit Exposure Groups**[a]	**Adjusted Odds Ratio (95% CI)**[c]	**p value**
Near Open Burn Pit and Open Burn Pit Duties	1.55 (1.39-1.72)	<.001
Near Open Burn Pit and Unknown Open Burn Pit Duties	1.12 (1.00-1.24)	0.04
Near Open Burn Pit and No Open Burn Pit Duties	1.13 (1.02-1.26)	0.02
Missing	1.11 (0.99-1.23)	0.07
**Days of Cumulative Exposure**[b]		
>448 days	1.46 (1.31-1.62)	<.001
290- 448 days	1.18 (1.06-1.32)	0.002
185- 289 days	1.06 (0.95-1.17)	0.33
1-184 days	1.05 (0.94-1.17)	0.37
Missing	1.11 (0.99-1.23)	0.07

[a]Model was based on exposure groups. Reference group was ‘Not Near Open Burn Pit and No Open Burn Pit Duties’.
[b]Model was based on cumulative exposure variable from the registry. Reference group was 0 days of open burn pit exposure.
[c]Odds ratios adjusted for pre-existing headache, age, sex, race and ethnicity, branch of service, and presence of traumatic brain injury.

© 2024 Sico JJ et al. *JAMA Network Open.*

eTable 3. Multinomial Logistic Regression Model Comparing Migraine and Other Headache Disorder with No Headache by Open Burn Pit Exposure Groups[a]

Open Burn Pit Exposure Groups	Adjusted Odds Ratio (95% CI)[b]	p value
**Non-Migraine Headache vs. No Headache**		
Near Open Burn Pit and Open Burn Pit Duties	1.43 (1.29-1.59)	<.001
Near Open Burn Pit and Unknown Open Burn Pit Duties	1.14 (1.03-1.27)	0.02
Near Open Burn Pit and No Open Burn Pit Duties	1.10 (0.99-1.23)	0.07
Missing	1.10 (0.99-1.23)	0.09
**Migraine vs No Headache**		
Near Open Burn Pit and Open Burn Pit Duties	1.81 (1.61-2.03)	<.001
Near Open Burn Pit and Unknown Open Burn Pit Duties	1.20 (1.06-1.34)	0.003
Near Open Burn Pit and No Open Burn Pit Duties	1.18 (1.05-1.33)	0.004
Missing	1.16 (1.03-1.31)	0.01

[a]Results are based on a single multinomial logistic regression model. The outcome had 3 levels and was defined as follows: migraine diagnosed by a medical provider, other headache disorder diagnosed by a medical provider, and no headache disorder. Those who had headache diagnoses in addition to migraine were considered to have migraine. Model was based on exposure groups. Reference group was 'Not Near Open Burn Pit and No Open Burn Pit Duties' and "No Headache".

[b]Odds ratios adjusted for age, sex, race and ethnicity, branch of service, and presence of traumatic brain injury.

© 2024 Sico JJ et al. *JAMA Network Open.*

eTable 4. Overall Headache Diagnosed by Near Open Burn Pit Exposure and Open Burn Pit Duties

**Open Burn Pit Exposure Groups**[a]	**Adjusted Odds Ratio (95% CI)**[b]	**p value**
Open Burn Pit Duties	1.40 (1.37-1.43)	<.001
Near Open Burn Pit	1.14 (1.04-1.25)	0.004
Unknown Open Burn Pit Duties	1.02 (1.00-1.05)	0.09
Missing Near Open Burn Pit	1.13 (1.03-1.23)	0.01

[a]Model was based on two exposure groups. Reference group for "Near Open Burn Pit" was 0 days of open burn pit exposure and the reference group for "Open Burn Pit Duties" was 0 days with open burn pit duties .
[b]Odds ratios adjusted for age, sex, race and ethnicity, branch of service, and presence of traumatic brain injury.

© 2024 Sico JJ et al. *JAMA Network Open.*

eTable 5. Migraine Diagnosed by Near Open Burn Pit Exposure and Open Burn Pit Duties

Open Burn Pit Exposure Groups[a]	Adjusted Odds Ratio (95% CI)[b]	p value
Open Burn Pit Duties	1.39 (1.35-1.43)	<.001
Near Open Burn Pit	1.15 (1.03-1.29)	0.01
Unknown Open Burn Pit Duties	1.00 (0.96-1.03)	0.88
Missing Near Open Burn Pit	1.13 (1.01-1.27)	0.03

[a]Model was based on two exposure groups. Reference group for "Near Open Burn Pit" was 'Not Near Open Burn Pit' and the reference group for "Open Burn Pit Duties" was 'No Open Burn Pit Duties'.
[b]Odds ratios adjusted for age, sex, race and ethnicity, branch of service, and presence of traumatic brain injury.

eTable 6. Overall Headache Diagnosed by Exposure Groups Excluding Those with Missing Open Burn Pit Exposure Information[a]

Open Burn Pit Exposure Groups[b]	Adjusted Odds Ratio (95% CI)[c]	p value
Near Open Burn Pit and Open Burn Pit Duties	1.60 (1.46-1.74)	<.001
Near Open Burn Pit and Unknown Open Burn Pit Duties	1.17 (1.07-1.28)	<.001
Near Open Burn Pit and No Open Burn Pit Duties	1.14 (1.04-1.25)	0.004

[a]Sensitivity analysis with the missing group removed.
[b]Model was based on exposure groups. Reference group was 'Not Near Open Burn Pit and No Open Burn Pit Duties'.
[c]Odds ratios adjusted for age, sex, race and ethnicity, branch of service, and presence of traumatic brain injury.

© 2024 Sico JJ et al. *JAMA Network Open.*

eTable 7. Migraine Diagnosed by Near Open Burn Pit Exposure and Open Burn Pit Duties Excluding Those with Missing Open Burn Pit Exposure Information[a]

Open Burn Pit Exposure Groups[b]	Adjusted Odds Ratio (95% CI)[c]	p value
Near Open Burn Pit and Open Burn Pit Duties	1.61 (1.44-1.80)	<.001
Near Open Burn Pit and Unknown Open Burn Pit Duties	1.15 (1.03-1.29)	0.01
Near Open Burn Pit and No Open Burn Pit Duties	1.15 (1.03-1.29)	0.01

[a]Sensitivity analysis with the missing group removed.
[b]Model was based on exposure groups. Reference group was 'Not Near Open Burn Pit and No Open Burn Pit Duties'.
[c]Odds ratios adjusted for age, sex, race and ethnicity, branch of service, and presence of traumatic brain injury.

© 2024 Sico JJ et al. *JAMA Network Open.*

eTable 8. Self-Reported Disabling Migraine by Open Burn Pit Exposure Groups

**Open Burn Pit Exposure Groups**[a]	**Adjusted Odds Ratio (95% CI)**[c]	**p value**
Near Open Burn Pit and Open Burn Pit Duties	1.93 (1.69-2.20)	<.001
Near Open Burn Pit and Unknown Open Burn Pit Duties	0.94 (0.82-1.08)	0.37
Near Open Burn Pit and No Open Burn Pit Duties	1.20 (1.05-1.37)	0.007
Missing	1.07 (0.94-1.23)	0.31
**Days of Cumulative Exposure**[b]		
>448 days	1.31 (1.15-1.50)	<.001
290- 448 days	1.19 (1.04-1.36)	0.01
185- 289 days	1.17 (1.02-1.34)	0.03
1-184 days	1.19 (1.04-1.36)	0.01
Missing	1.07 (0.94-1.23)	0.31

[a]Model was based on exposure groups. Reference group was 'Not Near Open Burn Pit and No Open Burn Pit Duties'.
[b]Model was based on cumulative exposure variable from the registry. Reference group was 0 days of open burn pit exposure.
[c]Odds ratios adjusted for age, sex, race and ethnicity, branch of service, and presence of traumatic brain injury.

© 2024 Sico JJ et al. *JAMA Network Open.*
