# Supplementary material for: Open Burn Pit Exposure in Headache Disorder and Migraine
Source: JAMA Netw Open. 2024 Sep 4;7(9):e2431522. doi: 10.1001/jamanetworkopen.2024.31522 (PMC11375476; doi:10.1001/jamanetworkopen.2024.31522)
Supplement: Supplement 2. — Data Sharing Statement [file jamanetwopen-e2431522-s002.pdf]

Data Sharing Statement

Sico. Open Burn Pit Exposure in Headache Disorder and Migraine. *JAMA Netw Open*. Published September 04, 2024. doi:10.1001/jamanetworkopen.2024.31522

Data
Data available: Yes
Data types: Deidentified participant data
How to access data: VHA Headache Centers of Excellence Administrative Cohort Data will be made available in accordance with VA Information Resource Center (VIReC) guidelines to eligible VA investigators. All data is to remain behind the VA firewall.
When available: With publication

Supporting Documents
Document types: Statistical/analytic code
How to access documents: VHA Headache Centers of Excellence (HCoE) Administrative Cohort Data code will be made available in accordance with VA Information Resource Center (VIReC) guidelines. Data related to open burn pit exposure cannot be directly shared by the HCoE. Please contact the Airborne Hazards and Bur Pit Centers of Excellence (AHBPCE) to apply for access to data elements from the Airborne Hazards and Open Burn Pit Registry.
When available: With publication

Additional Information
Who can access the data: Eligible VA investigators.
Types of analyses: Any purpose.
Mechanisms of data availability: After approval of a proposal and with a signed data access agreement.
Any additional restrictions: Data is to remain behind the VA firewall.
